# SICOB-endorsed national Delphi consensus on obesity treatment optimization: focus on diagnosis, pre-operative management, and weight regain/insufficient weight loss approach

**DOI:** 10.1007/s40519-023-01537-4

**Published:** 2023-02-10

**Authors:** Marco Antonio Zappa, Angelo Iossa, Luca Busetto, Sonja Chiappetta, Francesco Greco, Marcello Lucchese, Fausta Micanti, Geltrude Mingrone, Giuseppe Navarra, Marco Raffaelli, Delphi Expert Panel, Settimio Fabrizio Altorio, Luigi Angrisani, Claudio Arcudi, Fabrizio Bellini, Paolo Bernante, Rossana Berta, Esmeralda Capristo, Maria Grazia Carbonelli, Giovanni Casella, James Mariolo Casella, Lidia Castagneto Gissey, Maria Rosaria Cerbone, Franco Ciampaglia, Luigi Ciccoritti, Alessandro Contine, Giuseppe Currò, Rosella D’Alessio, Massimiliano De Palma, Daniela Delle Piane, Nino Di Benedetto, Nicola Di Lorenzo, Giovanni Fantola, Rahimi Farnaz, Mirto Foletto, Pietro Forestieri, Lucia Frittitta, Elisa Galfrascoli, Paolo Gentileschi, Cristiano Giardiello, Piero Giustacchini, Maria Paola Giusti, Ilenia Grandone, Caterina Guidone, Amerigo Iaconelli, Erminia Lembo, Silvana Leanza, Erminia Lembo, Giovanni Lezoche, Cesare Lunardi, Gennaro Martines, Bernardo Marzano, Emanuela Paone, Francesco Saverio Papadia, Federico Perrone, Luigi Piazza, Vincenzo Pilone, Pietro Pizzi, Mark Rice, Andrea Rizzi, Ferruccio Santini, Giuliano Sarro, Angelo Schettino, Nicola Tartaglia, Mauro Toppino, Antonella Usai, Maurizio De Luca

**Affiliations:** 1 Director of General Surgery Unit, Asst Fatebenefratelli-Sacco Milan, Milan, Italy; 2 Department of Medico Surgical Sciences and Biotechnologies Sapienza Polo Pontino, ICOT Hospital Latina, Latina, Italy; 3 Department of Medicine, University of Padua, Padua, Italy; 4 Obesity and Metabolic Surgery Unit, Department for General Surgery, Ospedale Evangelico Betania, Naples, Italy; 5 Director of Bariatric and Metabolic Surgery Unit, Istituto ospedaliero Fondazione Poliambulanza di Brescia, Brescia, Italy; 6 Director of General and Bariatric Surgery Unit, Santa Maria Nuova Hospital-Azienda Sanitaria Toscana Centro, Florence, Italy; 7 UOC Psychiatric and Psychologic School of Medicine, University Federico II of Naples, Naples, Italy; 8 Università Cattolica del Sacro Cuore, Rome, Italy; 9 Department of Medical and Surgical Sciences, Fondazione Policlinico Universitario A. Gemelli IRCCS, Rome, Italy; 10 Division of Diabetes and Nutritional Sciences, School of Cardiovascular and Metabolic Medicine and Sciences, King’s College London, London, UK; 11 Department of Human Pathology, University of Messina, Messina, Italy; 12 U.O.C. of Endocrine and Metabolic Surgery, Fondazione Policlinico Universitario Agostino Gemelli IRCCS and CREO, Università Cattolica del Sacro Cuore, Rome, Italy; 13 Director of General Surgery Unit Ospedali di Rovigo e di Trecenta, Trecenta, Italy

**Keywords:** Obesity treatment, Obesity pre-operative management, Weight regain approach, Insufficient weight loss approach, Italian Delphi consensus

## Abstract

**Purpose:**

Overweight and obesity affects 60% of adults causing more than 1.2 million deaths across world every year. Fight against involved different specialist figures and multiple are the approved weapons. Aim of the present survey endorsed by the Italian Society of Bariatric Surgery (SICOB) is to reach a national consensus on obesity treatment optimization through a Delphi process.

**Methods:**

Eleven key opinion leaders (KOLs) identified 22 statements with a major need of clarification and debate. The explored pathways were: (1) Management of patient candidate to bariatric/metabolic surgery (BMS); (2) Management of patient not eligible for BMS; (3) Management of patient with short-term (2 years) weight regain (WR) or insufficient weight loss (IWL); (4) Management of the patient with medium-term (5 years) WR; and (5) Association between drugs and BMS as WR prevention. The questionnaire was distributed to 65 national experts via an online platform with anonymized results.

**Results:**

54 out of 65 invited panelists (83%) respond. Positive consensus was reached for 18/22 statements (82%); while, negative consensus (s20.4; s21.5) and no consensus (s11.5, s17) were reached for 2 statements, respectively (9%).

**Conclusion:**

The Delphi results underline the importance of first-line interdisciplinary management, with large pre-treatment examination, and establish a common opinion on how to properly manage post-operative IWL/WR.

**Level of evidence V:**

Report of expert committees.

## Introduction

Obesity is a constantly growing, multifactorial, chronic, and recurrent disease worldwide. American Medical Association and other regulatory bodies recognized obesity as a disease in 2013 [1]. In Italy, based on a WHO report (2022) [2], 58.5% of the adult population (> 20 years old) was overweight and 19.9% was affected by obesity. Lifestyle change, anti-obesity medications (AOMs), and endoscopic procedures are non-surgical options to reduce weight and ameliorate related complications. Despite good results observed in the short term, durability represents a weak point of non-surgical treatment. A recently published population-based cohort study, Datalink, reported that among patients with clinically severe obesity, only 1 in 1290 men and 1 in 677 women could achieve normal weight using non-surgical means [3]. Wing et al. reported similar findings in their review, demonstrating that 80% of individuals who achieve a weight loss of 10% of their body weight will regain that weight within one year [4]. According to the current Food and Drug Administration (FDA) guidance, pharmacotherapy is approved for patients with a BMI ≥ 30 kg/m^2^ or ≥ 27 kg/m^2^ obesity-comorbidity, e.g., type 2 diabetes **(**T2D), hypertension, lipid disorders, obstructive sleep apnea, heart disease [5]. Orlistat, naltrexone/bupropion, and liraglutide are approved for weight loss management in Italy. Despite the logic of using medication to enhance weight loss, less than 3% of individuals who are living with obesity are undergoing treatment with prescription medication, a similar number of patients as in Italy [6–8]. Such low prescription rates are due to the lack of training in the science of obesity, the limited familiarity of AOMs, concern over their safety, lack of sufficient resources to support the patient, and biased attitudes toward obesity [6, 8]. Pharmacology represents the second line of obesity treatment currently and is explored, especially in the case of weight regain recidivism after bariatric surgery.

Since its introduction, bariatric metabolic surgery (BMS) has been explored in all the relative risks and benefits; at present, BMS represents the best choice to treat severe obesity and associated medical problems [9]. European Association for Endoscopic Surgery (EAES) Guidelines (2020), endorsed by the International Federation for the Surgery of Obesity and Metabolic Disorders—European Chapter (IFSO-EC), the European Association for the Study of Obesity (EASO), and the European Society for the Peri-operative Care of the Obese Patient (ESPCOP) support the recommendation to consider laparoscopic BMS for patients with BMI ≥ 40 kg/m^2^ and BMI ≥ 35–40 kg/m^2^ with associated medical problems comorbidities that are expected to improve with weight loss (*Strong recommendation*) or for patients with ≥ BMI 30–35 kg/m^2^ and T2D and/or arterial hypertension with poor control despite optimal medical therapy (*Strong recommendation*) [10]. Despite showing excellent long-term results and having recognized superiority compared with conservative treatment, BMS suffered by potential weight regain is not considered a surgical failure but is intrinsic to obesity defined as a chronic and recurrent disease [10]. Weight regains (WR) can affect 20–25% of patients after their nadir weight [11, 12]. Though some authors have proposed behavioral and biological mechanisms for WR [12], the pre-operative factors that predispose patients to significant WR remain undefined. Identifying these factors could improve the counseling of patients regarding the prevention of WR [12–14]. Moreover, revisional bariatric surgery (RBS) to manage WR and insufficient weight loss (IWL) may have higher complication and mortality rates compared to primary BMS [12, 15].

Clear diagnosis, psychological and nutritional support, pharmacological prescription, and BMS represent, at present, a comprehensive strategy to treat the chronic disease of obesity, but this strategy often lacks integrated cooperation. Because of the shared and common approach to optimizing the results of obesity treatment, the focus of the following research endorsed by the Italian Society of Surgery of Obesity and Metabolic Diseases (SICOB—Società Italiana di Chirurgia dell'OBesità e delle malattie metaboliche) will be on building consensus of national experts on the optimal management of the patient with obesity eligible or not for BMS, and to establish a consensus on WR/IWL management in the short and medium term to provide handling clinical guidelines for a clinician, daily approaching to obesity fight.

## Methods

Delphi method, a structured technique, is aimed at obtaining a consensus opinion from a panel of experts in areas wherein evidence is scarce, and opinion is important by repeated rounds of questionnaires [16, 17]. This process generally begins with an open-ended research question. The question is discussed among a group of content experts (steering committee) through an iterative process involving the sharing of opinions, professional experience, and scientific evidence. The process (question sourcing) leads to the development of a list of statements/items (Delphi questionnaire), which are then submitted to a broader panel of professionals to survey their level of agreement on the topics proposed (Delphi rounds of consensus) [16, 17]. The process is completed when feedback converges, providing no new elements of insight (reaching saturation). In the current study, the consensus process consisted of a one-step web-based Delphi method, which took place between May and July 2022. The survey was developed by a promoting group of eleven physicians [eight bariatric surgeons (DL.M, Z.M.A., I.A.; C.S., G.F., L.M., N.G., R.M.), one diabetologist (G.M.), one internal medicine physician (B.L.), one psychiatrist (M.F.)], identified as key opinion leaders (KOLs) in their respective fields in Italy (Steering Committee). The KOLs met to exhaustively analyze the published literature and discuss the unmet needs of the topic. Hence, the KOLs identified 22 statements highlighting a major need for clarification and debate, focused on the optimization of patients affected by obesity in different and multiple clinical situations. The explored pathways included the following:

1. Management of the patient candidate to BMS (3–6 months before);
2. Management of the patient not eligible for BMS;
3. Management of the patient who did not respond to BMS (IWL or report WR during two years post-surgery;
4. Management of the patient who has WR 5 years post-surgery; and
5. Association between pharmacological and BMS to improve outcomes and reduce WR.

Table 1 shows the 22 approved statements submitted to the Delphi process. In the absence of recognized criteria, the KOLs decided to establish the following IWL/WR definition:

**Table 1:** Statements approved by KOLs and submitted to Delphi evaluation

Area	Statement/Item
#1 Management of the patient candidate for bariatric surgery (3–6 months before)	Statement 1: In patient’s candidate to bariatric surgery preoperatively (3–6 months) is recommended to obtain weight loss: 1.1 Any amount 1.2 Minimum 5% 1.3 Minimum 10% Statement 2: Pre-operative weight loss helps to: 2.1 Improve surgical outcomes reducing related complications 2.2 Improve post-operative weight loss Statement 3: Adequate pre-operative screening should ever include evaluation for: 3.1 Endocrinopathies responsible for secondary obesity (e.g., thyroid disease and hypercortisolism) 3.2 Genetic syndromes causing severe obesity (e.g., Prader Willi syndrome) 3.3 Diabetes (T2DM) 3.4 Evaluation of glycemic compensation (if diabetes is known) 3.5 Therapy intensification if HbA1c > 7% 3.6 Dyslipidemias 3.7 OSAS (obstructive sleep apnea syndrome) 3.8 Overnight ventilatory treatment in patients with moderate-severe OSA 3.9 Estrogen therapy whose pre-operative suspension of at least one month reduce the thromboembolic risk 3.10 Diagnosis of micro /macronutrients (iron, folic acid,albumin) deficiencies 3.11 Diagnosis of vitamins D/B12 deficiency/insufficiency 3.12 Diagnosis of vitamins A/E/K deficiency / insufficiency 3.13 pre-operative supplementation of deficiency status Statement 4: Pre-operative weight loss must be achieved with any strategy (pharmacological/endoscopic / nutritional / psychological) Statement 5: Pharmacotherapy is helpful in achieving pre-operative weight loss Statement 6: The use of anti-obesity drugs in the pre-operative phase can improve surgical outcomes (% of medical / surgical complications) Statement 7: A thorough investigation aimed to gain significant surgical outcomes should always identify the presence of peculiar 7.1 eating behaviors: Grazing, Binge, Loss of Control Eating (LOC) and sweet eating 7.2 Eating Disorders: Binge Eating Disorder (BED) and Night Eating Syndrome (NES) Statement 8: The Very-Low-Calorie Diet (VLCD) / Very-Low Ketogenic Diet (VLKD) protocols (400/800 kcal / d) and Low-Calorie Diet (LCD) (800–1200 kcal / d) for a pre-operative period of 2–12 weeks represents a strategy for weight loss and liver volume reduction Statement 9: Reduction of ≥ 10% in weight, of 3 kg of fat or 5% of excess weight represent the target of the pre-operative nutritional strategy Statement 10: Nutritional treatments, including pharmacological ones, presents low clinical risks and didn’t compromise significantly (in terms of timing) the pre-operative process
#2 Management of the patient not eligible for bariatric surgery	Statement 11: The following types of patients are never eligible for bariatric surgery: 11.1 Patients with obesity older than 60 years with high anesthesiologic risk 11.2 Patients with psychiatric disorders such as: schizophrenia, psychosis, addiction, obsessive–compulsive disorder, borderline disorder, personality disorders, bulimia 11.3 Patients in psychopharmacological treatment of which it is not possible to evaluate post-operative plasma levels to avoid psychic decompensation conditions 11.4 Patients who refuse nutritional supports 11.5 Patients showing a significant weight increase during the pre-operative supportive process Statement 12: The perioperative protocol must include interdisciplinary assessments of risk / benefit and protocols for evaluating the reversibility of contraindications Statement 13: Age > 70 years in absence of high pre-operative risks does not represent itself a criterion for exclusion from surgical therapy Statement 14: In patients suffering from obesity not suitable for surgical procedures the transient (e.g., Bioenterics Intragastric Balloon-BIB) or potentially reversible (endoscopic sleeve) endoscopic strategies represents a valid alternative Statement 15: In patients suffering from obesity not suitable for surgical procedures the nutritional strategies (any type) represent a safe and effective alternative Statement 16: patients suffering from obesity not suitable for surgical procedures nutritional strategies (any type) together with anti-obesity drugs represents a safe and effective alternative Statement 17: In patients suffering from obesity not suitable for surgical procedures the pharmacological strategies per se (even in the absence of a nutritional strategy) represents a safe and effective alternative Statement 18: Recent scientific evidence has shown that among the pharmacological therapies for weight control, GLP-1 receptor agonists show greater efficacy Statement 19: Any strategy (endoscopic/pharmacological/nutritional) cannot ignore a psychological support strategy
#3 Management of the patient who in the 2 years post-surgery did not respond to bariatric surgery (loss of insufficient weight-IWL) or reports weight regain (WR)	Statement 20: In patient with obesity who in the 24 months post-surgery did not respond to surgery due to insufficient weight loss (IWL) or because it reports significant weight regain (WR) (according to previously specified criteria) we can state that: 20.1 is necessary an adequate and broad psychological and nutritional framework as first phase of treatment 20.2 any type of intervention requires an excellent micronutrients control 20.3 the prescription of pharmacotherapy represents a valid strategy of treatment 20.4 The comorbidities control in presence of IWL / WR makes unnecessary a nutritional strategy 20.5 Endoscopic revision is a good strategy intervention 20.6 Revisional surgery is a good intervention strategy
#4 Management of the patient who in the 5 years post bariatric surgery has a weight regain	Statement 21: In the patient with obesity who in the 5 years post bariatric surgery reports a significant weight regain we can state that: 21.1 is necessary an adequate and broad psychological and nutritional framework as first phase of treatment 21.2 an adequate and broad psychological / psychiatric framework is necessary to evaluate the causes 21.3 any type of intervention requires an excellent micronutrients control 21.4 the prescription of pharmacotherapy represents a valid strategy treatment 21.5 The comorbidities control in presence of WR makes unnecessary a nutritional strategy nutritional strategy 21.6 Endoscopic revision is a good strategy intervention 21.7 Revisional surgery is a good intervention strategy
#5 The pharmacological association in the post-operative period can improve the outcome of surgery	Statement 22: Scientific evidence shows how the use of anti-obesity drugs after surgery can facilitate weight loss and / or stop weight regain

*IWL*: weight loss < 20% of the initial weight or that does not shift the patient to a class of obesity different from the initial one or that does not lead to control clinically significant metabolic complications.

*Significant WR*: any weight regained by the nadir that lends itself to the value or is very close to the initial value (first evaluation) with a detrimental effect on the quality of life or involving clinically inadequate control of metabolic complications.

Thereafter, the KOLs defined and validated the statements before Delphi analysis (Table 1). After approval, the questionnaire was distributed to 65 national experts in bariatric surgery and in a multidisciplinary obesity approach via an online platform with anonymized results.

### Selection criteria for expert panelists

The panelists were invited to indicate their level of agreement or disagreement on each statement using a 5-point Likert scale, scored from 1 to 5 (1, strongly disagree; 2, disagree; 3, agree; 4, mostly agree. 5, strongly agree). The experts were selected based on the selection criteria listed in Table 2. Results were expressed as a percentage of respondents who scored each item as 1 or 2 (disagreement) or as 3, 4, or 5 (agreement). A positive consensus was reached in the case of > 66% agreement, a negative consensus in the case of < 66% disagreement, and the consensus was not reached when the sum for disagreement or agreement was < 66% [18]. After getting relevant literature on the topic, the KOLs decided whether to proceed to the second round in a dedicated meeting, for the statements on which consensus could not be achieved. Descriptive statistics were used to summarize the results. The current study is based on a survey that neither involves the participation of human subjects nor patient data management and does not aim to modify the current clinical practice of participants. Consequently, this study did not require any ethical approval.

**Table 2:** Inclusion criteria of Delphi respondents

	Experience	SICOB Affiliation	Distribution	Sexual distribution
Bariatric surgery expert	At least 5 years of experience in bariatric surgery	Members	Homogeneous distribution of the whole national territory	Unfeasible given the small number of female representatives in surgery
Multidisciplinary team expert	Involved in the multidisciplinary team of a SICOB center with at least 5 years of experience in management of obese patients	Not mandatory	Homogeneous distribution of the whole national territory	At least 40% female representation (ideally 50%)

## Results

### Degree of consensus in the Delphi process

In the first round of the Delphi survey, there were 54 (83%) respondents out of 65 invited panelists. Thirty-seven (68.5%) were male while 17 (31.5%) were female respondents. In terms of age distribution, 6 (11%) people were 30–35 years old, 3 (5.5%) were 3–40 years old, 7 (13%) were 41–45 years old, 13 (24%) were 46–50 years old, 7 (13%) were 51–55 years old, 4 (7%) were 56–60 years old, 9 (17%) were 61–65 years old, 2 (4%) were 66–70 years old and 3 (5.5%) were 70 years old. Panelists participated homogenously from the whole country, specifically 18 (33%) from the North, 21 (39%) from the Centre, and 15 (28%) from the South/Islands of Italy. The respondent group included: 40 general surgeons’ experts in bariatric surgery, six endocrinologists, one specialist in internal medicine, five dietitians/nutritionists, and two psychologists. Regarding the clinical experience, 12 respondents (22.2%) reported between 21 and 25 years of experience in obesity treatment, 9 (16.6%) between 11–15 and 5–10 years, 7 (13%) between 26 and 30 years, 6 (11.1%) between 16 and 20 years, 5 (9%) between 36 and 40 years, 4 (7%) between 31 and 35 years, and two (3.7%) respondents had > 40 years of experience.

**Fig. 1:**
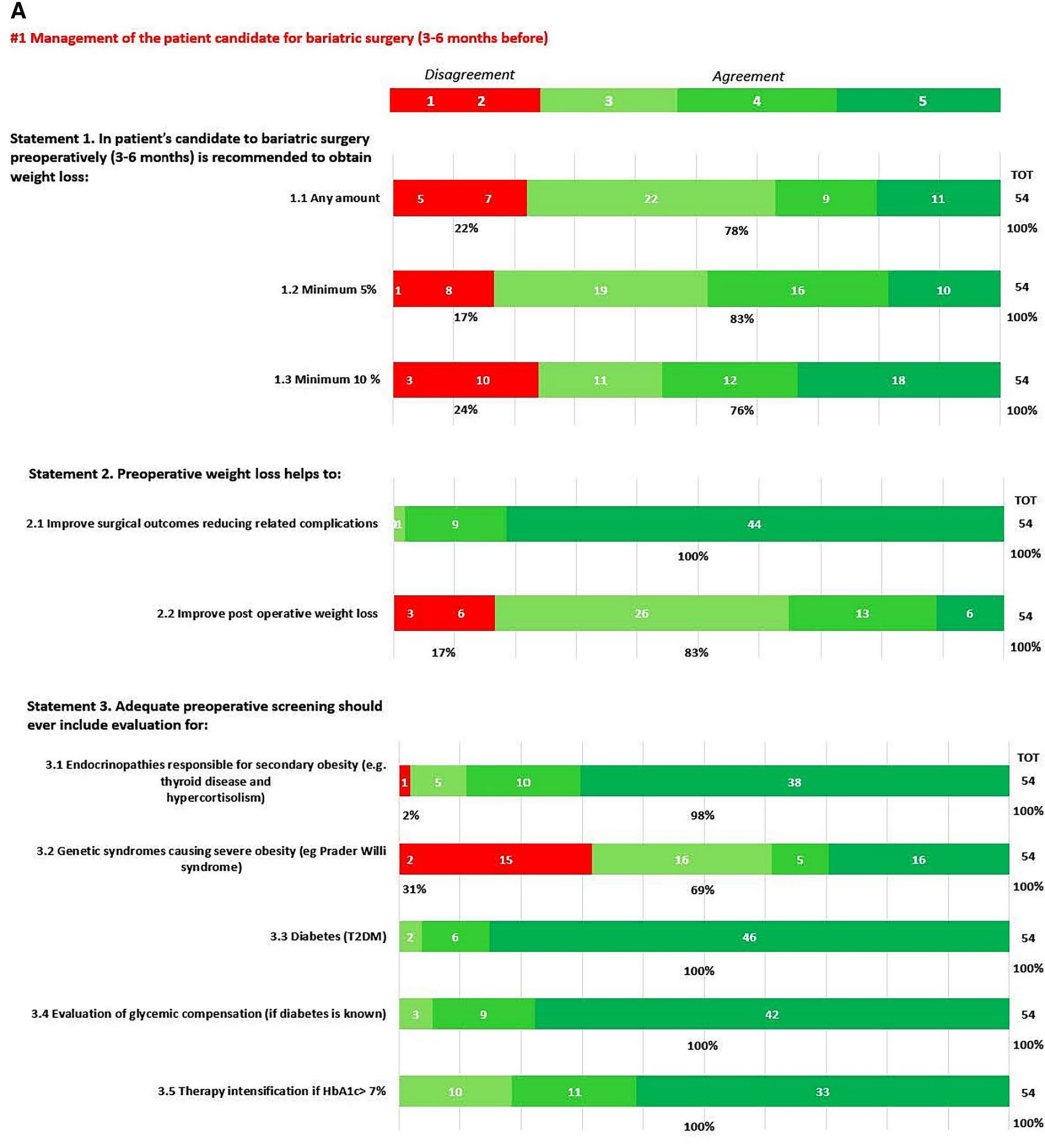

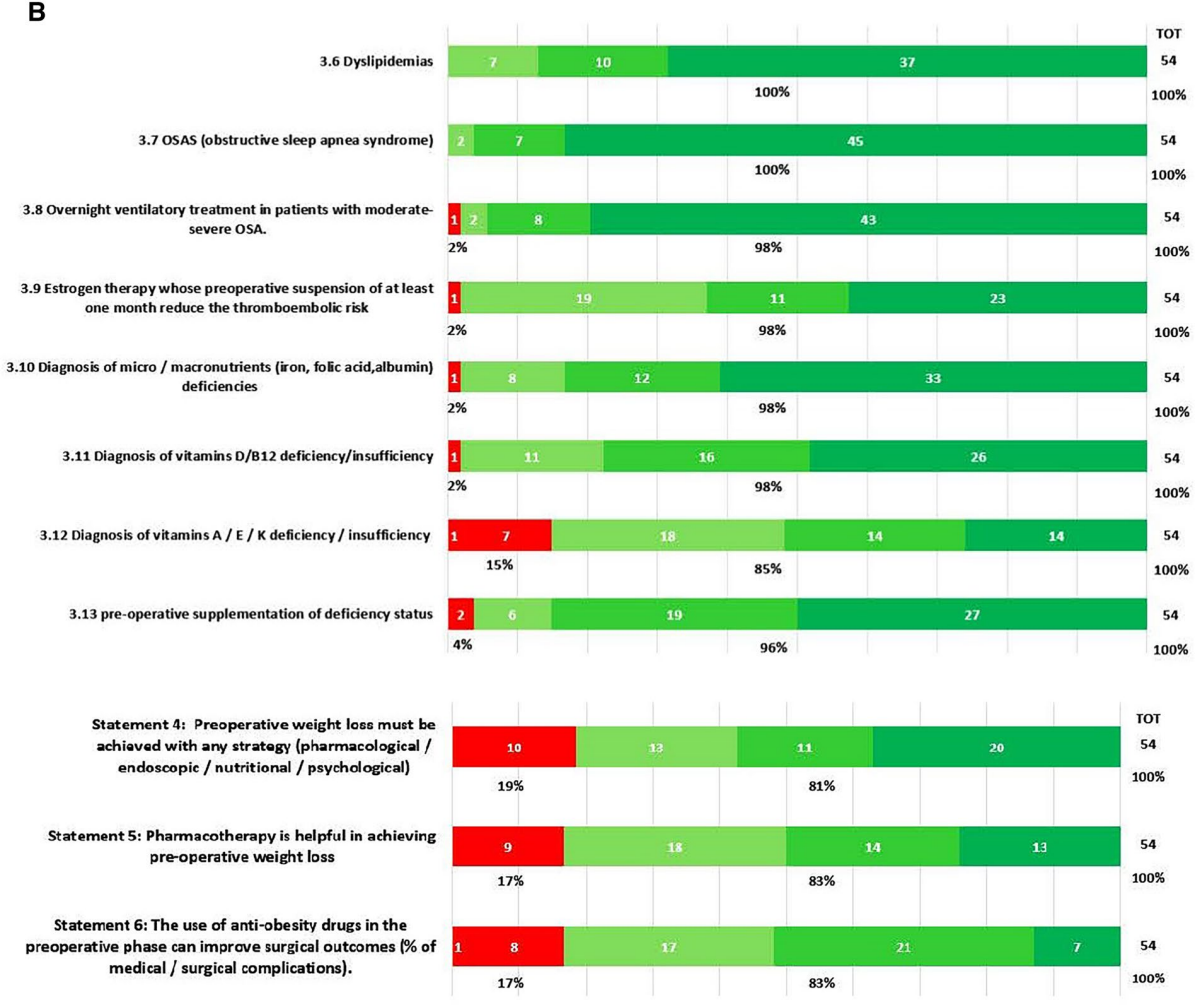

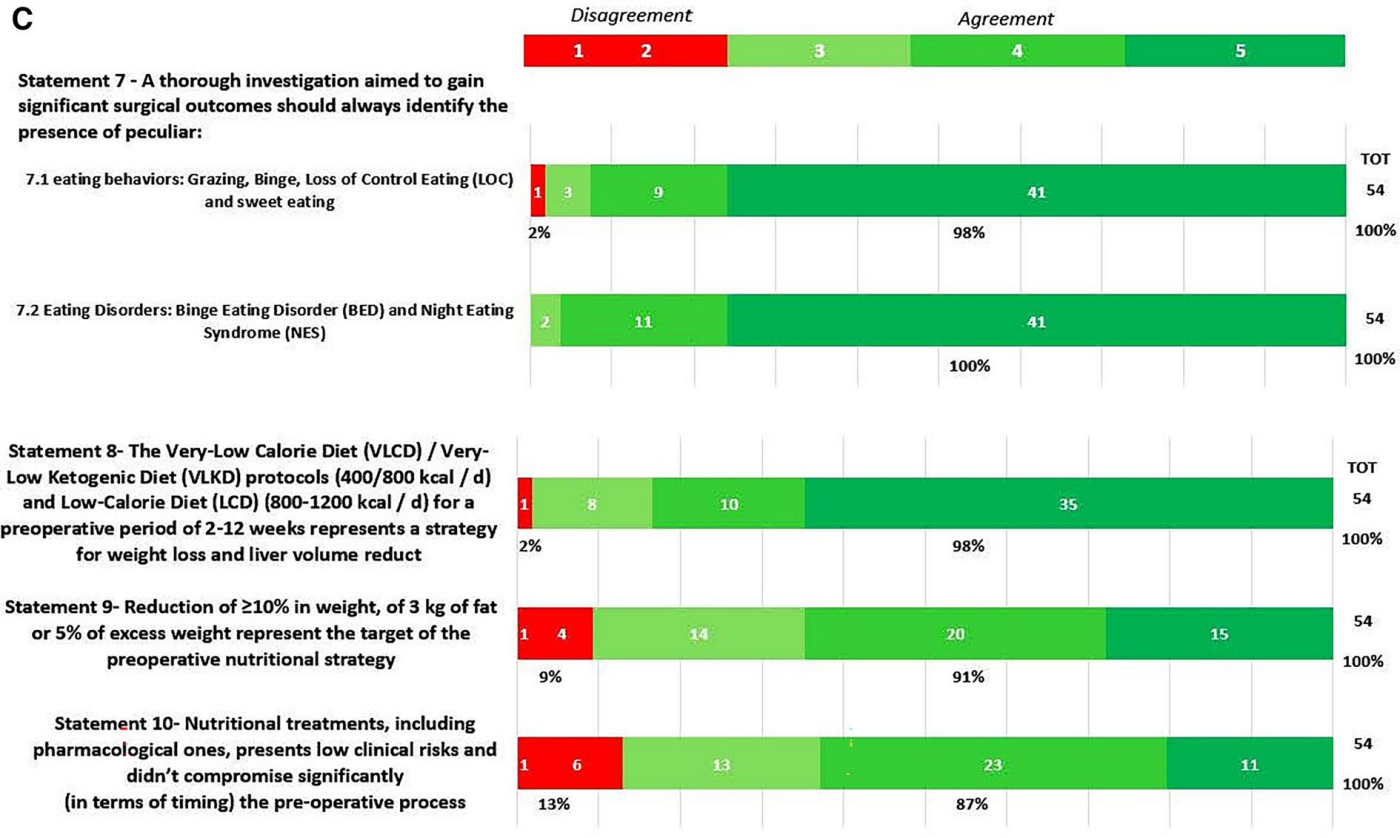
**A**, **B**, **C**: Delphi results on Area: Management of the patient candidate to BS(3–6 months before); In green statement reaching positive consensus

**Fig. 2:**
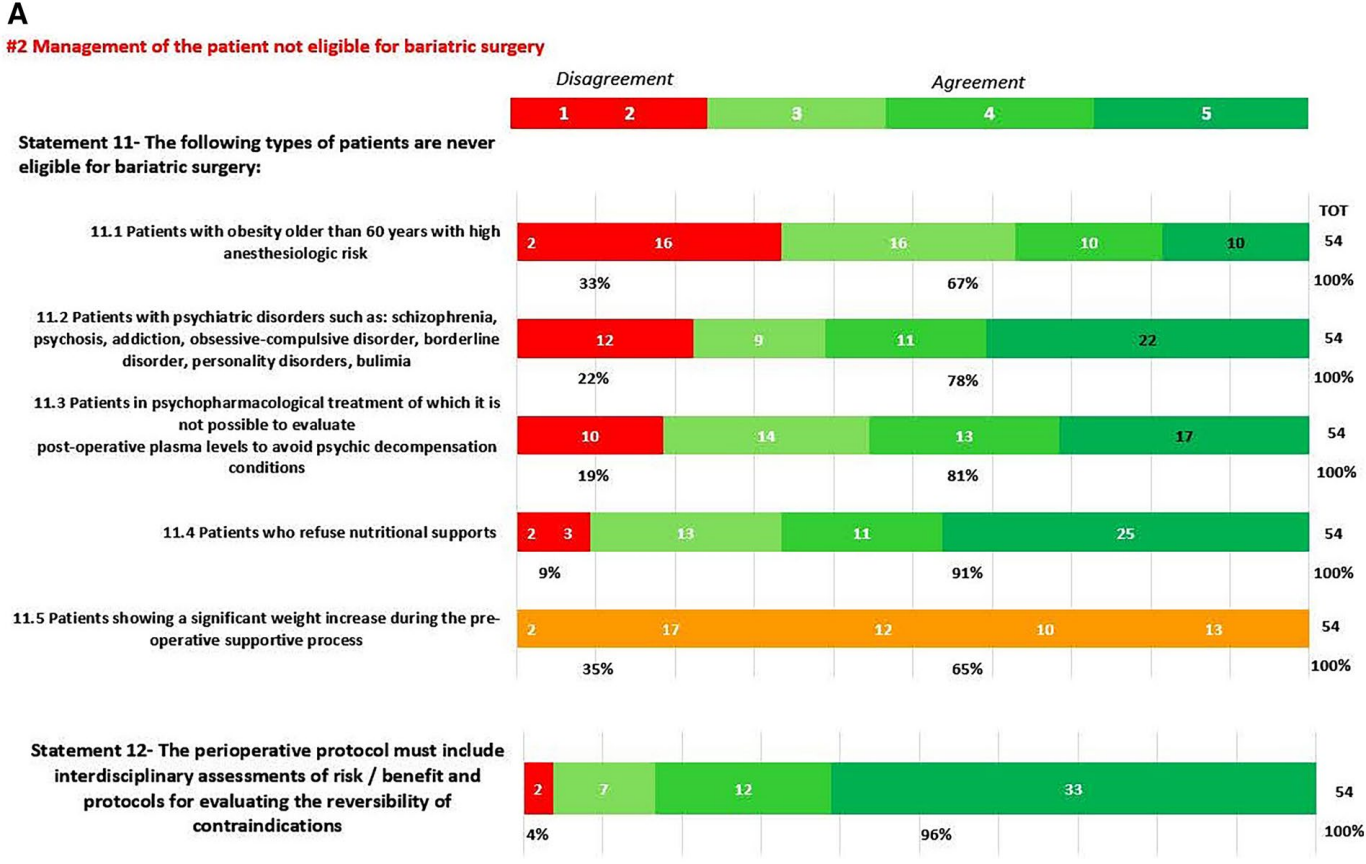

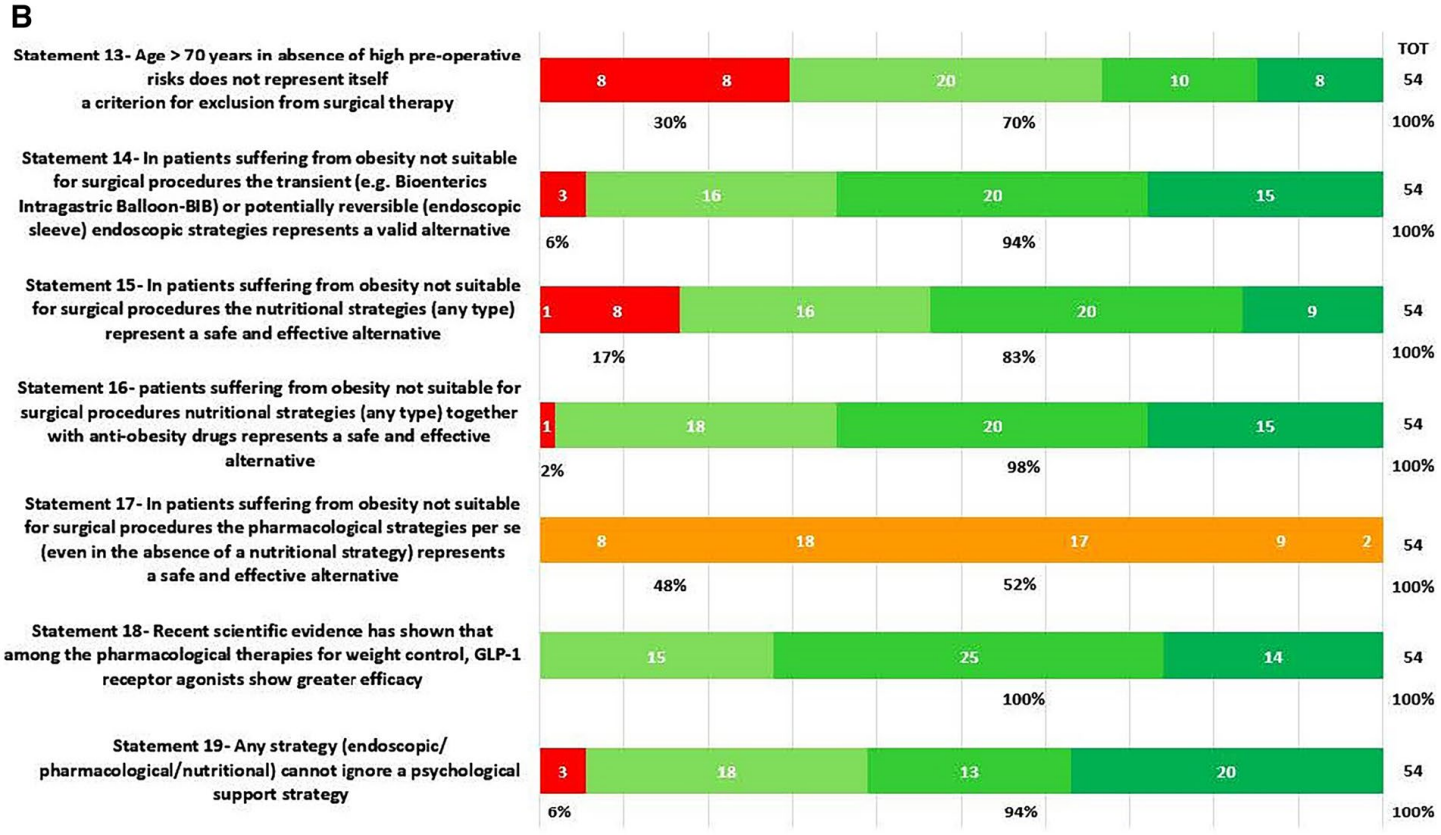
**A**, **B**: Deplhi results on Area: Management of the patient not eligible for bariatric surgery In green statement reaching positive consensus, in yellow statement without consensus

**Fig. 3:**
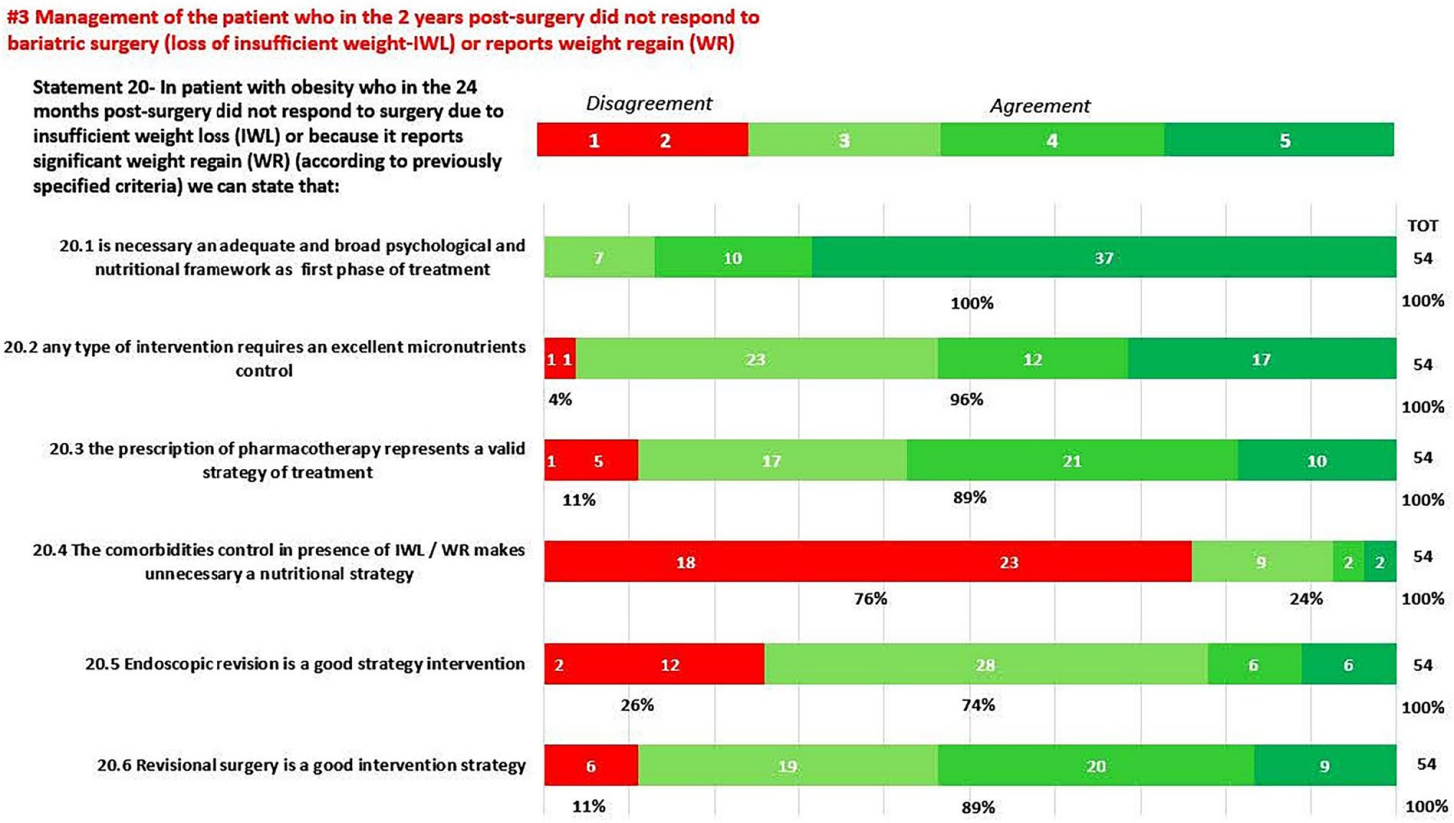
Deplhi results on Area: Management of the patient who in the 2 years post-surgery did not respond to bariatric surgery (loss of insufficient weight—IWL) or reports weight regain (WR); In green statement reaching positive consensus, in red statement with negative consensus

**Fig. 4:**
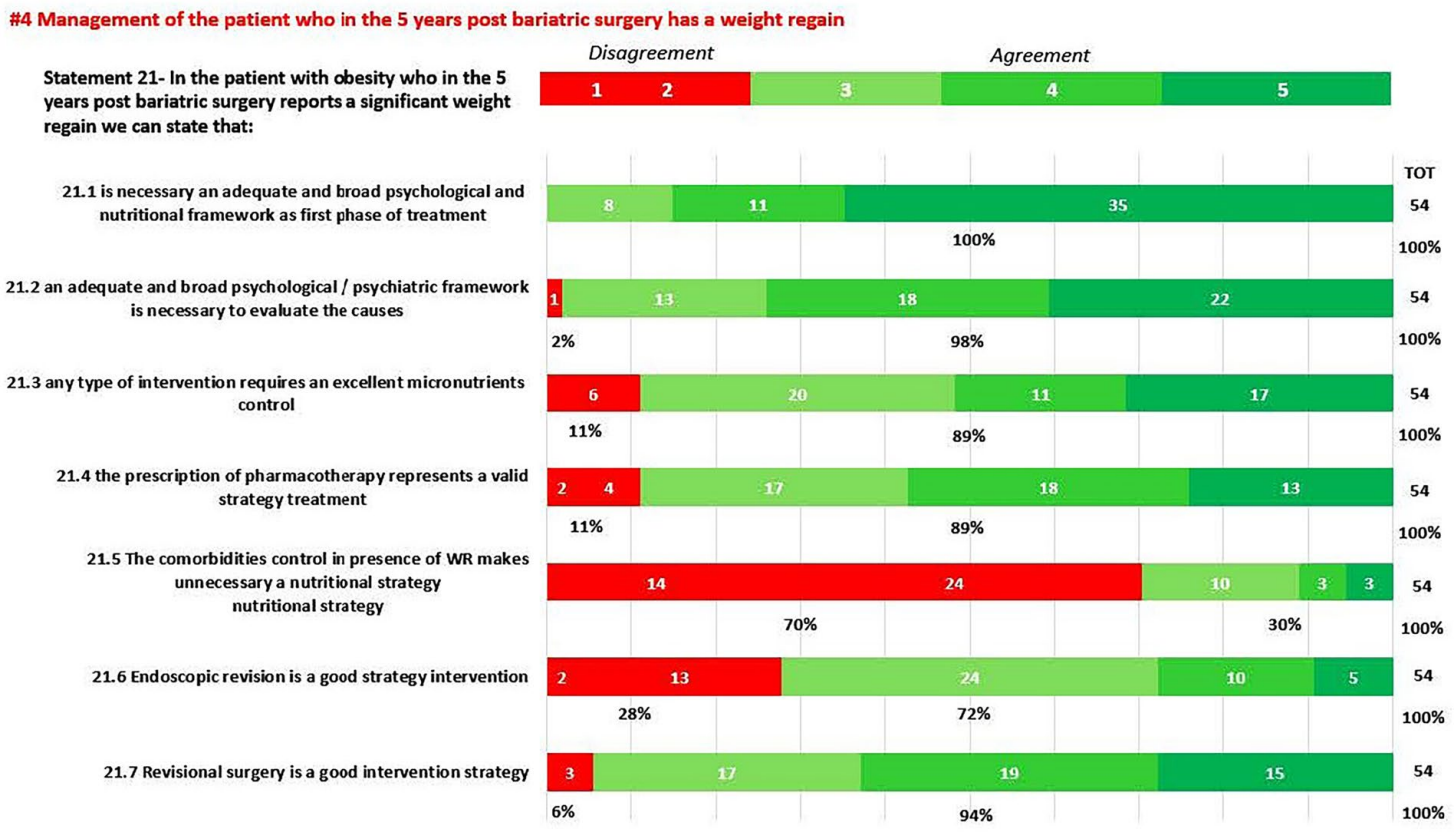
Deplhi results on Area: Management of the patient who in the 5 years post bariatric surgery has a weight regain; In green statement reaching positive consensus, in red statement with negative consensus

**Fig. 5:**
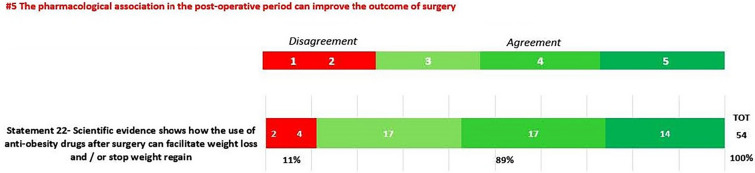
Delphi results on Area: The pharmacological association in the post-operative period can improve the outcome of surgery; In green statement reaching positive consensus

In round 1, positive consensus was reached for 18 statements (82%) (s1.1, 1.2, 1.3, 2.1, 2.2, 3.1, 3.2, 3.3, 3.4, 3.5, 3.6, 3.7, 3.8, 3.9, 3.10, 3.11, 3.12, 3.13, 4, 5, 6, 7.1, 7.2, 8, 9, 10, 11.1, 11.2, 11.3, 11.4, 12, 13, 14, 15, 16, 18, 19, 20.1, 20.2, 20.3, 20.5, 20.6, 21.1, 21.2, 21.3, 21.4, 21.6, 21.7, 22) out of 22, while negative consensus (s20.4, 21.5) and no consensus (s11.5, 17) were reached for 2 statements, respectively (9%). After dedicated meetings, the steering committee decided not to perform a second round for the two statements without consensus. Figures 1A,B–C, 2A–B, 3, 4 and 5 report graphically the results of each of the 22 statements.

## Discussion

This paper, endorsed by the SICOB, represents the first Delphi method study on the optimization of obesity treatment to establish a commonly approved pathway to treat a common and life-threatening disease. We divided the research into five areas evaluating the pre-operative time, possible contraindications, the post-operative time with a specific focus on WR and IWL, and the association between anti-obesity drugs and surgery.

### Pre-operative weight loss (Statements 1, 2, 4, 5, 6, 8, 9, 10)

- With statements 1, particularly s1.1, s1.2, and s1.3, the respondents agreed that weight loss is recommended prior to BMS, but the consensus achieved for any weight loss target probably reflects an absence of accepted, precise goals. Pre-operative weight loss remains debatable. Currently, most of the relevant guidelines provide no clear indication about pre-operative weight loss [10, 19, 20]. Guidelines agreed that a period of identifiable medical management is necessary for all patients prior to BMS and that it is also necessary to assess patients’ motivation and willingness to adhere to follow-up programs, but pre-operative weight loss is neither mentioned in the indication for BMS nor in the pre-operative evaluation. Nevertheless, different studies exist in the current literature, which underlines the importance of pre-operative weight loss to achieve technical operability [21, 22].

The KOL agree with the responders to consider weight loss, at least weight maintenance, as part of the pre-operative nutritional re-assessment and a strong sign of patient’s motivation.

Regarding the method to achieve pre-operative weight loss (s4,5,6), the respondents think that all the available supporting strategies to lose weight must be adopted before bariatric surgery. Pre-operative weight loss can be obtained with several regimens, such as low-calorie diets (LCD) (800–1200 kcal/day), very low-calorie diets (VLCD) (600 kcal/day), very-low-calorie ketogenic diet (VLCKD) (400/800 kcal/d) and the question of which method provides the best results in terms of weight loss and patients’ compliance, tolerance and acceptance remain debatable [23, 24]. The National Institute for Health and Care Excellence (NICE) [25] recommends pharmacological treatment for weight loss maintenance in addition to a reduced calorie diet and optimal physical exercise [26–28]. A systematic review confirmed that VLCD led to a significant weight loss of 2.8 to − 14.8 kg together with liver size reduction (− 5–20% of the initial volume) [29]. A more recent study comparing the effect of VLCD, and LCD showed that VLCD was more effective in reducing total body weight [30]. VLCKD demonstrated similar VLCD weight loss results, but with a significant liver volume reduction (5.8 vs. 4.2%) [30]. The efficacy of liraglutide on weight loss has been demonstrated by the Safety and Clinical Adiposity–Liraglutide Evidence (SCALE) trials. The SCALE obesity and pre-diabetes double-blind RCT demonstrated significantly higher weight loss with liraglutide vs placebo (− 5.8% for liraglutide 3.0 mg versus − 1.5% with placebo) at 56 weeks [31]. The evidence on multiple advantages related to pre-operative nutritional treatments (in terms of intra/perioperative complications and post-operative adherence) and the short-term duration (3–12 weeks) makes this pre-operative approach safe, largely approved, and not significantly increase the time spent on the waiting list. Because there is no proven evidence of a single strategy’s absolute advantage, the choice of the type of pre-surgical weight loss approach (s8, 9, 10) should reside on the patient’s characteristics and local expertise.

Importantly, the post-operative degree of weight loss is positively influenced by pre-operative measures. Therefore, re-education based on nutritional consultation should be considered for patients undergoing bariatric surgery as recommended by EAES guidelines (Strong recommendation) [10].

### Pre-operative interdisciplinary evaluation (Statement 3, 7)

Pre-operative work-up needs a complete evaluation of physical, metabolic, and psychologic statuses. In statement 3.1 agreement (98%) as established by the American Society for Metabolic and Bariatric Surgery (ASMBS) Guidelines update 2020, the endocrine evaluation is strongly recommended before performing BMS [19]. Similarly, the SICOB guidelines in 2016 established that the endocrinopathies responsible for secondary obesity must be rightly evaluated pre-surgery for an adequate selection of patients (level of evidence: 4; degree of recommendation c) [20]. Consensus remains elusive on genetic screening in obese patients. The respondent agreed that genetic syndromes should be tested before bariatric surgery (agreement 69%) because several studies have reported variable and sometimes unpromising results in patients with syndromic/monogenic syndromes. ASMBS updated guidelines state that case-by-case decisions should be made based on specific historical and physical findings (Grade D) [19]; the KOL together with the results of the Delphi round emphasized that these findings could be useful to predict scarce or moderate bariatric surgery results but should be recommended only in patients with clear clinical suspicion. Diabetes (s 3.3., s3.4, s3.5; 100% agreement), dyslipidemias screening (s3.6; 100% agreement), and Obstructive Sleep Apnea Syndrome (OSAS) screening (s3.7; 100% agreement) must be properly diagnosed, treated, and balanced based on multiple intersociety recommendations [10, 19, 20]. Micro/macronutrients (s3.10, 3.11, 3.12, and 3.13, all receiving agreement), should be balanced prior to performing surgery to reduce the risk of post-operative deficiencies [32]. Aasheim et al. [33] analyzed the vitamin status of 110 patients affected by severe obesity compared with 58 normal-weight subjects. Patients with obesity had significantly lower concentrations of vitamins A, B6, C, 25-hydroxyvitamin D, and lipid-standardized vitamin E. Similarly, Van Rutte et al. in their study of 200 patients affected by severe obesity demonstrated that 38% had low serum iron, 24% had low serum folate, 11% had low serum vitamin B12, and 81% had hypovitaminosis D [34]. Furthermore, Peterson et al. demonstrated a frank deficiency of vitamin D and iron in 71.4% and 36.2% of 58 BS candidates, respectively [35]. Together with the clinical/metabolic status, the psychosocial behavioral situation should always be investigated (s7.1, s7.2, s11, s19 agreement 98–100%). ASMBS updated guidelines require a formal psychosocial behavioral evaluation to be performed by a qualified behavioral health professional before a bariatric procedure (Grade C; BEL 3) [19]. Any patient considered for a bariatric procedure with a known or suspected psychiatric illness, or substance abuse or dependence should first undergo a formal mental health evaluation (Grade C; BEL 3) [19]. Under the national SICOB guidelines of 2016 [20], anxiety and/or depression (endogenous or reactive to the condition of obesity), eating disorders (binge eating disorder [BEG], night-eating syndrome, bulimia, eating disorders with loss of control [LOC]), and personality disorders were considered relative contraindications susceptible to re-evaluation after adequate therapy (level of evidence1.2; degree of recommendation A).

### Contraindications to bariatric surgery (Statements 11, 12, 13)

High anesthesiologic risk represents an absolute contraindication to proceed independently of the age factor that does not represent a per se contraindication (s11.1) as reported by several studies even in patients aged < 70 years. High anesthesiologic risks include factors such as severe heart failure, unstable coronary artery disease, end-stage lung disease, active cancer treatment, and portal hypertension [36]. Furthermore, because these procedures are performed under general anesthesia, any contraindication to general anesthesia would also be a contraindication for these surgeries. Evaluation of risk versus benefit is performed on a case-by-case basis, but outcomes analysis of patients aged > 70 years using the American College of Surgeons National Surgical Quality Improvement Program (ACS-NSQIP) database reported that the overall rate of morbidity and mortality are significantly higher in this patient’s category [37]. Rates of several adverse were increased in patients aged > 70 years undergoing Roux-en-Ygastric bypass (RYGB), indicating SG a preferred procedure for fragile patients [37]. The increased rates of morbidity and mortality observed for patients with impaired functional status support consideration of functional status when evaluating pre-operative risk (s13). Regarding psychiatric disorders (s11.2), the EAES (2020) guidelines [10] suggest that most mental disorders (mood, anxiety, bipolar disorder, eating disorders, etc.) might be considered a contraindication if severe and/or undertreated [10]. Similarly, according to the Italian SICOB guidelines 2016 [20], non-compensated bipolar disorder is generally considered an absolute contraindication to BMS, both for its symptomatologic characteristics and the difficulty in stabilizing the pharmacological treatment in post-surgery. Similarly, decompensated schizophrenia and psychosis are absolute contraindications to BMS (level of evidence: 2, grade of recommendation B) [20]. In patients undergoing psychopharmacological treatment (s 11.3), it is mandatory to check how surgical choice, restrictive vs malabsorptive, and weight loss impact the drugs plasma level. This monitoring should include an assessment of the accuracy of the patient’s diagnosis and the need for the psychotropic agent, documentation of a pre-surgery plasma level of many drugs, and an assessment of the level of symptomatology of their illness. From the nutritional point of view (s11.4), refusing nutritional support represents an absolute contraindication because it impairs the physician–patient relationship, a cornerstone of medical practice.

Based on Delphi s11.5, considering pre-operative significant weight increase the contraindication for surgery (absolute and unmodifiable) was one of the two statements that eluded consensus. After KOLs re-evaluation, the steering group decided not to perform a second Delphi round because in line with the decision of non-agreement. A weight increase during the pre-operative process certainly represents a temporary surgical contraindication but it cannot be considered absolute because far from the re-education (that needs time and, sometimes, mistakes) and the welcoming attitude of bariatric centers that know that surgery, at present, represents the best and durable treatment for severe obesity and its comorbidities.

### Non-surgical weight loss strategies (Statements 14, 15, 16, 17, 18, 19)

Regarding the non-surgical strategies available at the end of the process of indication/contraindication (S14) multiple endoscopic treatments have been proposed and offered to those patients who refuse surgery or are not suitable for surgery. Procedure selection depends on costs, ability to pay, patient’s risk profile, center’s experience, patient’s preferences, and comorbidities to treat [38]. The KOLs agreed with the respondent about the safety of all the nutritional strategies in patients unsuitable for surgical/invasive procedures. Current Food and Drug Administration (FDA) guidelines have approved pharmacotherapy for patients with a BMI ≥ 30 kg/m^2^ or ≥ 27 kg/m^2^ in presence of associated medical problems [25, 39]. Despite using medication to enhance weight loss (s16),  < 3% of obese individuals are being treated with prescription medication [7]. A recent metanalysis of randomized controlled trials (RCT) [27] revealed a significant reduction in body weight with orlistat, lorcaserin, phentermine plus topiramate, naltrexone plus bupropion and liraglutide compared to placebo (all p < 0.00001). Although there were no head-to-head trials amongst these five drugs, the authors documented that the weight reduction abilities of these drugs in descending order are: phentermine plus topiramate > liraglutide (3.0 mg) > naltrexone plus bupropion > lorcaserin = orlistat [27]. Regarding comorbidities effects nevertheless, liraglutide 3.0 mg should be the preferred agent in obese type 2 diabetes subjects [27, 31]. In Italy, at present, orlistat, liraglutide, and naltrexone plus bupropion are approved as anti-obesity drugs. GLP-1 agonist (liraglutide) has undergone multiple trials for efficacy and safety [31, 40–44]. It is available in Italy and the agreement of 100% is probably based on comparative experience against the other two available drugs (orlistat and naltrexone plus bupropion) in terms of weight loss. Importantly, higher costs, as well as tolerability, remain significant barriers in prescribing these medications. Pharmacological strategies required a mandatory nutritional strategy to optimize weight loss results, for this reason, S17 (that did not reach consensus) was not submitted to the second round.

### IWR/WR management in short- and medium-term follow-up (Statement 20, 21)

Despite the excellent long-term results and the recognized superiority compared with conservative treatment, bariatric surgery suffered by potential weight regain, is not considered as a surgical failure but is sometimes intrinsic to the obesity definition as a chronic/recurrent disease with a WR percentage of 25% [11–14]. Several mechanisms are advocated in IWL/WR; wrong primary surgical indications, hormonal/metabolic balance, dietary non-adherence, mental health problems and physical inactivity [11–14]. With statement 3, the KOLs would explore the attitude of clinicians toward patients demonstrating IWL/WR in a short-term (24 months) period after any kind of bariatric surgery. All the professional figures working in bariatric surgery settings know that the mandatory first step in those cases is represented by a re-assessment of pre-operative conditions to re-establish a new starting point (s20.1; 100% agreement). The offers are behavioral cognitive therapy, remote acceptance-based behavioral intervention, lifestyle counseling together with dietary counseling with a dietitian, and structured dietary intervention. Regarding nutritional conditions, one of the mechanisms advocated in IWL/WR after bariatric surgery must be considered for follow-up discontinuation [14]. This attitude can cause an insufficient nutritional status, particularly in terms of micronutrient levels. As for the pre-operative process, the re-evaluation needs to reset all the conditions and consequently, the excellent nutritional balance (s20.2; 96% agreement) of patients’ needs to be established. The IWL/WR even in the presence of comorbidities control needs a mandatory nutritional strategy because weight recidivism is associated with the deterioration of the quality of life and the reappearance or worsening of obesity-associated comorbidities [45, 46] while bariatric surgery recognizes as goal adequate weight loss together with comorbidities control/amelioration/cure. With the s20.4 disagreement, the experts and the KOLs need to educate patients about the needing to control their weight, especially to avoid sensitive WR, then the need for nutritional and psychological constant support to not waste the results obtained in obesity-related comorbidities. In terms of management, several AOMs (s20.3) have been used in conjunction with lifestyle modifications to decrease hunger, promote satiety, and halt the WR after BS. The research found that, among 319 patients with WR or inadequate WL post-RYGB or LSG, 54%, 30.3%, and 15% of the sample lost ≥ 5%, ≥ 10%, and ≥ 15% of their total body weight (TBW), respectively, using medications with favorable outcomes using topiramate [47]. Another study on young adults (*n* = 37) with WR showed that 54.1%, 34.3%, and 22.9% of the samples lost ≥ 5%, ≥ 10%, and ≥ 15% of their postsurgical weight, respectively, [48]. An evaluation of liraglutide 3 mg among 117 patients who undertook RYGB, laparoscopic adjustable gastric banding (LAGB), or LSG revealed that patients achieved statistically significant WL (− 6.3 ± 7.7 kg) seven months and stable at 1 year, regardless of the type of primary surgery [49]. Generally, there are few studies on the use of prescription weight loss medications to treat WR or IWL and are primarily retrospective, and no studies were available to determine the best medication/s or timing of introduction of the medication. Different operative, endoscopic or surgical management approaches have been proposed (s20.5, s20.6) as good strategies in IWL/WR management. Revision of BS is indicated to resolve surgical complications, A less invasive endoluminal approach [50, 51], if safe and effective, could be a reasonable option offering a more favorable risk profile in these patients, but is not always feasible. Summing up, revisional surgery is largely proposed worldwide and is continuously getting popular but it is hard to establish a common decisional-making process [10]. In the extant literature, no RCTs have documented the effects of various revisional surgeries on WR/IWL for failed LAGB, LSG, and RYGB; hence, the question “what is the suitable type of revisional surgery for WR/IWL in terms of better WL and lower complications" remains addressed.

With the series of statement 4, KOL want to explore the attitude of clinicians toward patients demonstrating WR in a medium-term period (5-year FU) after any kind of bariatric surgery and particularly, the KOL want to try to compare different attitude compared with short-term IWL/WR management explored in statement 20 series. This statement series did not differ in agreement/disagreement balance compared with the short-term series and the clinicians seem at the end, to follow the same rules in WR management with an interdisciplinary approach (first line) to carry out behavioral/psychological causes followed by several opportunities (nutritional, pharmacological, endoscopic/surgical) based on the grade of WR, type of patients (age, risks, comorbidities relapse) and personal experience/expertise. Because of so large variability in approach to revisional treatment, EAES 2020 guidelines establish a position statement that “no evidence-based criteria for indication to revisional bariatric/metabolic surgery are available to date” and conclude that the clinical decision to proceed with revisional bariatric/metabolic surgery be based on a complete multidisciplinary assessment of the patient, as recommended for the primary procedure [10].

### Future approach (Statement 22)

With s22, we explore the perception that anti-obesity drugs immediately after surgery can facilitate weight loss and receive a sustained agreement. This topic is under exploration and currently seems to offer a promising amelioration of post-operative results. Thakur et al. [52] in a randomized, double-blind, placebo-control study evaluated 23 patients submitted to LSG and randomized to receive liraglutide (*n* = 12) or placebo (*n* = 11). Patients in liraglutide group had % EWL of 58.7 ± 14.3 as compared with 44.5 ± 8.6 (*p* = 0.043) in placebo group at 24 weeks. All patients with diabetes or pre-diabetes had a resolution of dysglycemia in the liraglutide group as compared with 50% in the placebo group [52]. Despite these limitations, the current study is an initial, single-center experience that has the potential to open a window to a novel research field, focusing on optimization and long-term maintenance of post-operative results.

At present several are the option to optimize the obesity treatment but with this Delphi the study group has establish a common expert consensus. The results summarize that, commonly, the expert facing daily the obesity are following strict rules on pre-operative screening management and weight loss strategy and are managing the eventual IWL/WR by critical decision-making process even in absence on accepted specific guidelines.

## Conclusion

The current study represents the first Delphi consensus SICOB-endorsed on optimization process in obesity treatment with multiple focuses on different aspects of management. The Delphi results highlight the importance of interdisciplinary management, with large pre-treatment examination, as first-line and offer significant elements on how experts are facing WR and IWL with multiple lines of treatment (pharmacological, endoscopic, surgical). The present Delphi-mediated consensus could represent the first step to build recommendations specifically in not explored field of obesity treatment.

What is already known on this subject?

- Bariatric surgery represents the most accepted and durable therapy of obesity and clear is the process of pre-operative management established by the national/international guidelines
- It remains controversial how the clinicians commonly approach to some specific situations such as pre-operative weight loss, comorbidities amelioration and post-operative identification and cure of specific complications (e.g., weight regain/ insufficient weight loss)

What does this study add?

- The study represents the basis of national guidelines (first Delphi endorsed by the Italian Society of bariatric Surgery) on commonly interdisciplinary approved management of obesity in every treatment’s phase: pre-operative, post-operative (short term and long term), and with a specific focus on contraindications and non-operative management.

## Data Availability

Data are fully reported into the manuscript based on the paper type (Delphi).
